# Theoretical and experimental study of the pH-dependent interaction of amino acids and MFI-type zeolite

**DOI:** 10.1186/1758-2946-5-S1-O11

**Published:** 2013-03-22

**Authors:** Kai Stueckenschneider, Juliane Merz, Victor Milman, Gerhard Schembecker

**Affiliations:** 1 Department of Biochemical and Chemical Engineering, TU Dortmund University, Dortmund, 44227, Germany; 2 Accelrys Ltd, Cambridge, CB4 0WN, UK

## 

The separation and purification of peptides and proteins using chromatography can make up more than half the amount of the total purification costs of a biotechnological process [1]. The development of cost-efficient processes still suffers from the lack of understanding on a molecular level of adsorption mechanisms of biomolecules on materials surfaces.

To explore the power of computer science in supporting experimentally based process development, we applied a combined force field approach with subsequent Density Functional Theory (DFT) calculations. As a first step, we examined the adsorption of the amino acids glycine (gly), l-alanine (ala) and l-lysine (lys) on MFI-type zeolite (MFI). Experimental data from adsorption isotherms and Isothermal Titration Calorimetry (ITC) have proved the adsorption to be strongly pH-dependent. Therefore, we applied the amino acids in their protonated and neutral state in order to simulate low and medium pH values in our computational study. A T33 cluster containing all potential binding regions was used as a model of MFI.

Initial geometries for DFT calculations were prepared with force field methods in order to speed up the search in conformational space. Low energy adsorption sites were determined using Adsorption Locator and Forcite modules of Materials Studio 6.0 (Accelrys Inc.). Subsequently, the structures were optimized with DMol^3 ^code (Accelrys Inc.) using the generalized-gradient approximation (GGA) with the Perdew-Burke-Ernzerhof (PBE) exchange-correlation functional and the double numerical plus polarization (DNP) basis set.

Calculated energies strongly mirror the adsorption affinities derived from experimental adsorption isotherms: (lys_low_pH _>> ((ala > gly)_low_pH _~ lys_medium_pH_)) >> (ala > gly)_medium_pH_. Furthermore, calculated adsorption energy ratios between protonated amino acids agree very well with the enthalpy ratios derived from low pH ITC experiments: ([ala:gly] = 1.12_exp _= 1.14_calc_), ([lys:ala] = 1.72 _exp _= 1.75 _calc_), ([lys:gly] = 1.92_exp _= 1.97_calc_). Additionally, the adsorption mechanisms of neutral and protonated amino acids were found to be qualitatively different. Neutral amino acids bind via neutral amino group and proton transfer to the active site in zeolite, whereas protonated amino acids bind via carboxyl group.

These results illustrate that a judiciously designed combination of atomistic modeling methods can be used as a reliable first step in the design of cost-efficient industrial processes.
